# Interrogative suggestibility in the elderly

**DOI:** 10.1371/journal.pone.0241353

**Published:** 2020-11-16

**Authors:** Silvia Biondi, Cristina Mazza, Graziella Orrù, Merylin Monaro, Stefano Ferracuti, Eleonora Ricci, Alberto Di Domenico, Paolo Roma

**Affiliations:** 1 Department of Human Neuroscience, Faculty of Medicine and Dentistry, Sapienza University of Rome, Rome, Italy; 2 Department of Neuroscience, Imaging and Clinical Sciences, G. d’Annunzio University of Chieti-Pescara, Chieti, Italy; 3 Department of Surgical, Medical, Molecular & Critical Area Pathology, University of Pisa, Pisa, Italy; 4 Department of General Psychology, University of Padova, Padova, Italy; 5 Department of Psychological, Health, and Territorial Sciences, G. d’Annunzio University of Chieti-Pescara, Chieti, Italy; University of Auckland, NEW ZEALAND

## Abstract

Interrogative suggestibility (IS) describes the extent to which an individual behavioral response is affected by messages communicated during formal questioning within a closed social interaction. The present study aimed at improving knowledge about IS in the elderly (aged 65 years and older), in particular about its association with both emotive/affective and cognitive variables. The sample (*N* = 172) was divided into three groups on the basis of age: late adult (aged 55–64, *N* = 59), young elderly (aged 65–74, *N* = 63), and elderly (aged 75 and older, *N* = 50). Cognitive (i.e., Kaufman Brief Intelligence Test-2, Rey Auditory Verbal Learning Test), emotive/affective (i.e., Rosenberg Self-Esteem Scale, Marlowe–Crowne Social Desirability Scale, Penn State Worry Questionnaire) and suggestibility measures (i.e., Gudjonsson Suggestibility Scale-2) were administered. In order to identify differences and associations between groups in IS, cognitive and emotive/affective variables, ANOVAs tests and Pearson’s correlations were run. Furthermore, moderation analyses and hierarchical regression were set to determine whether age, cognitive and emotive/affective variables predicted IS components (i.e., Yield and Shift). Finally, machine learning models were developed to highlight the best strategy for classifying elderly subjects with high suggestibility. The results corroborated the significant link between IS and age, showing that elderly participants had the worst performance on all suggestibility indexes. Age was also the most important predictor of both Yield and Shift. Results also confirmed the important role of non-verbal intelligence and memory impairment in explaining IS dimensions, showing that these associations were stronger in young elderly and elderly groups. Implications about interrogative procedures with older adults were discussed.

## Introduction

In 1919, McDougall [1] defined suggestion as “a process of communication resulting in the acceptance with conviction of the communicated proposition in the absence of logically adequate grounds for its acceptance”. More recently, suggestibility is described as “a peculiar state of mind which is favourable to suggestion” [2]. Specifically, it has been described as the tendency to accept messages communicated during an interview in a way that influences one’s behavior and answers [3]. Starting from Sidis’s above quote [2], Hilgard [4] clarified that “suggestion” refers to an influential communication, while “suggestibility” refers to individual differences in responding to suggestions under comparable circumstances.

Interrogative suggestibility (IS) is a specific kind of suggestibility that has been defined by Gudjonsson and Clark [3] as “the extent to which, within a closed social interaction, people come to accept messages communicated during formal questioning, as a result of which their subsequent behavioral response is affected” (p.4). It is important to note that IS is a manifold construct with cognitive (e.g., verbal and non-verbal IQ, memory), social, and interpersonal (e.g., anxiety, social desirability, self-esteem) aspects [5, 6]. To improve knowledge about IS, researchers have studied its association with IQ [7–11], verbal communication [12–14], anxiety [15–18], depression [19, 20], social desirability [21], and self-esteem [22, 23]. All of these variables have been found to correlate with suggestibility, validating the IS model [3]. Concerning the relationship between IS and IQ, Gudjonsson [11] and Tully and Cahill [10] reported a negative correlation between these constructs, finding low scores on IQ variables to be associated with higher scores on suggestibility. Gudjonsson [18] also analyzed the relationship between anxiety and IS, finding that high levels of trait anxiety were associated with high levels of suggestibility. Furthermore, in the study of Baxter, Jackson, and Bain [23], all IS indexes were found to be significantly higher in participants scoring low in self-esteem.

IS plays a significant role in explaining the reliability of eyewitnesses—particularly, whether distortions caused by misleading questions affect eyewitness memory and testimony [3, 24]. This is an important construct to study in legal contexts, since eyewitness testimony is one of the most important sources of information in a trial [25]. Due to the obvious challenges relating to children’s recollection and their tendency to report fewer details to forensic interviewers [5], IS has been studied in children [12–14] and adolescents [9, 26], with some studies comparing IS in children to that of adults [7, 8, 27]. Other studies [7, 8, 27–32] have investigated IS in adults, while very few have analyzed IS in the elderly. In addition to there being an increasing number of senior citizens across nations [33], the elderly are also remaining actively involved in society for longer than they were in the past, and they are thus more likely to appear in legal contexts as victims or eyewitnesses to a crime. Therefore, although the elderly are at high risk for victimization and fraud—considering their impaired memory and increased susceptibility to leading questions and misinformation during interviews [34–39]—to the best of our knowledge, only three studies have investigated IS in this population [28–30].

Polczyk and colleagues [30] recruited 109 participants and divided them into two groups: 66 young adults (*M* age = 22.3, *SD* = 3.3, range: 18–35) and 43 older adults/elderly (*M* age = 64.1, *SD* = 9.5, range: 49–88). They then compared these groups on IS, as measured by the Gudjonsson Suggestibility Scale Version 2 (GSS-2) [31, 40–42]. The results showed that older participants scored higher on the GSS-2 Yield scale, but not the Shift scale. Furthermore, memory performance, as assessed by the Wechsler Memory Scale (WMS) [43] and the Memory Assessment Clinics Self-Rating Scale (MAC-S) [44, 45], seemed to have little influence on Shift scores, but were significant predictors of Yield scores.

Mueller-Johnson and Ceci [29] submitted 113 participants—62 college students (*M* age = 20.2, *SD* = 1.12, range: 18–20) and 51 elderly participants (*M* age = 76.4, *SD* = 7.85, range: 65–93)—to various relaxation techniques, such as body massage and aromatherapy. Afterwards, they provided misleading information to half of each group and compared participants’ recollection in repeated interviews. In doing so, they administered the Logical Memory subscale of the Wechsler Memory Scale (WMS) [43], the Vocabulary subscale of the Wechsler Adult Intelligence Scale Revised (WAIS-R) [46], the NEO-Five Factor Inventory (NEO-FFI) [47, 48], the Memory Functioning Questionnaire (MFQ) [49, 50], the GSS [31, 40–42], and the Gudjonsson Compliance Scale (GCS) [51]. The results highlighted an overall tendency for older participants to be more easily influenced by suggestive questions than younger participants, but no statistics were reported in relation to the GCS, “due to space restrictions” [29].

Dukala and Polczyk [28] compared 42 young adults (*M* age = 23, *SD* = 2.77; range: 16–29) with 41 young elderly people (*M* age = 66.82, *SD* = 2.17; range: 64–74). The 83 participants were randomly assigned to two experimental conditions that were differentiated according to interviewer behavior: friendly versus abrupt. They administered the GSS-2 [31, 40–42], the Memory Assessment Clinics Self-Rating Scale (MAC-S) [44, 45], the Mini-Mental State Examination (MMSE) [52], and a questionnaire requiring 5-point (1–5) Likert scale ratings on 18 aspects of the interviewer’s manner. The results showed that, when assigned to the first condition, young elderly people scored higher than young adults on the Yield scale and scored similarly on the Shift scale. However, when the interviewer behaved in an abrupt/unfriendly manner, young elderly participants scored higher on the Shift scale compared to young adults. Therefore, the second experimental condition seemed to increase uncertainty and reduce the memory of young elderly people, demonstrating in turn a relationship between age and suggestibility. Self-appraisal of memory was related to both age and interviewer behavior, and there was a tendency for elderly subjects to change answers after receiving negative feedback. These findings suggest that a friendly manner should be adopted when questioning elderly witnesses.

However, the samples of these studies were relatively young (*M* age = approximately 65 years) and not split into age classes. Furthermore, none of the three studies reported above engaged in a deep analysis of the ways in which IS variables might correlate with emotive/affective measures to further influence suggestibility [17, 18, 21, 22].

The main purpose of the present research was to promote knowledge about IS in older persons (aged 65 years and older)—and, in particular, the way in which IS is influenced by age—paving the way for a deeper conversation about the use of interrogative procedures with older adults. Furthermore, the study aimed at investigating the associations between IS and both emotive/affective and cognitive variables.

Specifically, following the results of the quoted research, we hypothesized:

1. elderly participants would show the worst performance on GSS-2 indexes, with higher scores on Yield and Shift scales and lower scores on Immediate Recall and Delayed Recall scales;
2. late adult, young elderly, and elderly groups would show significant negative correlations between Yield scores and cognitive variables, such as verbal and non-verbal intelligence (as measured by the KBIT-2 V and NV), memory (IR and DR RAVLT) and Immediate Recall (IR) and Delayed Recall (DR) scales of GSS-2;
3. late adult, young elderly, and elderly groups would show significant correlations between Shift scores and emotive/affective variables: positive between Shift scores, worry (as measured by PSWQ) and social desirability (MCSDS); and negative between Shift scores and self-esteem (SES).
4. age groups (late adult, young elderly, and elderly) would moderate the relationship between cognitive variables (KBIT-2 V and NV; IR and DR RAVLT; IR GSS-2 and DR) and Yield scores, as well as the relationship between emotive/affective variables (PSWQ, MCSDS, and SES) and Shift scores. All the relationships will be stronger for young elderly and elderly groups;
5. age and cognitive variables (KBIT-2 V and NV; IR and DR RAVLT; IR GSS-2 and DR) would significantly affect Yield scores as well as age and emotive/affective variables (PSWQ, MCSDS, and SES) would significantly affect Shift scores among all age groups.
  To increase replicability, the present research included behavioral prediction as well as statistical analysis. This served to verify that the proposed model was capable of predicting behavioral outcomes. Use of 10-fold cross-validation reduces variance in the model performance estimation [53, 54].
6. Finally, we used machine learning (ML) to validate a model to predict whether a subject had high or low suggestibility (defined as high vs. low GSS-2 Total Suggestibility score, high vs. low Shift score, or high vs. low Yield score), using cognitive and emotive/affective variables as predictors.

## Materials and methods

### Participants

The participants were 200 volunteers who participated in the study for a small reward (European breakfast in a cafe). The sample was comprised of 100 men and 100 women who were all European and aged 55–90 years. They were recruited from senior citizen centers (i.e., centers offering daily individual and group recreational activities for elderly people) based in Rome, Florence, Naples, and L’Aquila. The inclusion criteria were: (a) no history of neurological or psychological/psychiatric disorder, (b) an MMSE [51] score equal or superior to the age corrected score of 27, and (c) no major life events (e.g., grief or car accident) experienced in the previous 6 months. Data were collected from March 2016 to September 2018 by two interviewers—psychology graduates who had been formally trained in administering the GSS-2. One interviewer recruited subjects in Rome and Naples, while the other recruited in Florence and L’Aquila. No differences between the results on IS obtained by the two researchers were observed. Twenty-eight participants (14%) were excluded from the analysis for one or more of the following reasons: (a) an MMSE score below 27 (*N* = 21), (b) one or more major life events experienced (*N* = 1), and (c) a history of neurological or psychological/psychiatric disorder (*N* = 6). The remaining 172 participants (*M* age = 68.49; *SD* = 8.69; 94 women and 78 men) comprised the three research groups: late adult (aged 55–64 years), young elderly (aged 65–74 years), and elderly (aged 75–86 years). An overall linear relationship between years of education and age group was found, although a statistically significant difference was observed only between the late adult group and the other two groups (Table 1). The three groups did not differ in gender [χ^2^ (2) = .033, *p* = .983]. The study was carried out with written informed consent from all subjects, in accordance with the Declaration of Helsinki. It was approved in 2015 by the local ethics committee (Board of the Department of Human Neuroscience, Faculty of Medicine and Dentistry, Sapienza University of Rome).

**Table 1:** Descriptive statistics of the three groups.

	Late adult	Young elderly	Elderly
	55–64 years	65–74 years	75–85 years
	(*N* = 59)	(*N* = 63)	(*N* = 50)
**Age**_**years**_	59.64 (2.79)	69.10 (3.01)	78.14 (2.60)
**Education**_**years**_	11.20 (3.82) ^a^	8.56 (3.59) ^b^	7.02 (2.50) ^b^

*Note*: For each line, different letters indicate a significant difference between columns; F(2, 169) = 21.40, *p* = <.001.

### Materials and procedures

The test administration was split into two sessions. In the morning, participants completed individually: (a) the consensus form, (b) the anamnestic questionnaire, (c) the cognitive measures (MMSE, KBIT-2, and RAVLT), and (d) the emotive/affective measures (SES, MCSDS, and PSWQ). In the afternoon, they were administered the GSS-2. During the 50-minute interim, participants engaged in the senior citizen center’s regularly scheduled afternoon snack break. The MMSE was used only as an inclusion/exclusion criterion and was not considered in the data analyses.

#### Cognitive measures

*Mini Mental State Examination (MMSE)* [52]. The MMSE is a 30-item questionnaire that is used extensively in clinical and research settings to measure cognitive impairment. Commonly, it is used in medical and clinical settings to screen for dementia. Administration of the test takes 5–10 minutes, and it examines functions including registration (the ability to repeat named prompts), attention and calculation, recall, language, the ability to follow simple commands, and orientation [55]. In the present study, a cut-off of 27 was used to distinguish subjects with suspected cognitive impairment [56].

*Kaufman Brief Intelligence Test-2 (KBIT-2)* [57]. The KBIT-2 is a brief intelligence test that takes approximately 15–20 minutes to administer. It measures an individual’s verbal (via vocabulary subtests) and non-verbal (via matrices subtests) intelligence. The verbal subtests measure crystallized ability and the non-verbal subtests measure fluid reasoning. There are three scores for the KBIT-2 test: Verbal (V), Non-Verbal (NV), and Composite IQ. The present study used the first two measures (i.e., KBIT-2 V, KBIT-2 NV) of the Italian version of the test [58], which showed good internal consistency: verbal IQ α = 0.91; non-verbal IQ α = 0.93.

*Rey Auditory Verbal Learning Test (RAVLT)* [59]. The RAVLT is a well-recognized measure of a subject’s ability to encode, combine, store, and recover verbal information in different stages of immediate memory. The test is designed as a list-learning paradigm, whereby a list of 15 words (List A) is presented and the subject is asked to recall as many words from the list as possible (testing immediate recall [IR]). After five repetitions of free recall, a second “interference” list of words (List B) is administered and the participant is asked to recall as many words from List B as possible. After the interference trial, the participant is asked to recall words from List A (testing delayed recall [DR]). After a 20-minute delay, the participant is asked to again recall words from List A. The present study used the Italian version of the test [60], which showed sufficient internal consistency (α = .76). Raw scores were corrected for age and years of education.

#### Emotive/affective measures

*Rosenberg Self-Esteem Scale (SES)* [61]. The SES is a 10-item scale that investigates global self-worth by measuring both positive and negative feelings about oneself. The scale has been described as unidimensional and all of the items are rated on a 4-point Likert scale ranging from 1 (*strongly agree*) to 4 (*strongly disagree*). The SES is widely used for evaluating self-esteem, with high scores suggesting high self-esteem. Example items include: “On the whole, I am satisfied with myself” and “I take a positive attitude toward myself.” Several studies have shown the SES to demonstrate good psychometric properties [62]. The present study used the Italian version of the SES, which was shown to have good internal consistency (α = .84) [63].

*Marlowe–Crowne Social Desirability Scale (MCSDS)* [64]. The MCSDS consists of 33 true/false items selected to have socially desirable content and a low probability of occurrence. Participants respond to each item by indicating whether it is true or false. An example item is: “I never hesitate to go out of my way to help someone in trouble.” High scores reveal that the subject tends to present him/herself in an unrealistically favorable manner. The MCSDS scale scores were shown to demonstrate an internal reliability coefficient of .88 in a sample of undergraduate students, and high concurrent validity, as established through correlations with the MMPI validity scales [61]. The present study used the Italian version of the MCSDS, which was shown to demonstrate good internal consistency (α = .81) [65].

*Penn State Worry Questionnaire (PSWQ)* [66]. The PSWQ consists of 16 items regarding problematic worries that individuals might apply to themselves. Example items include: “Once I start worrying, I cannot stop” and “I do not tend to worry about things.” All items are rated on a 5-point Likert scale ranging from 1 (*not at all like me*) to 5 (*very much like me*). The PSWQ has undergone extensive psychometric evaluation and is generally accepted as a reliable and valid measure of problematic worry [67, 68]. The present study used the Italian version of the MCSDS, which was demonstrated to show good internal consistency (α = .84) [69].

#### Suggestibility measure

*Gudjonsson Suggestibility Scale-2 (GSS-2)* [31, 40–42]. The GSS-2 entails a short interrogation procedure, during which the participant is asked to listen to a brief story and answer questions that mimic social pressure. In the present study, the scale was administered as follows [42]: first, the interviewer read a short story out loud (180 words with 40 significant details) at a fairly slow pace. The subject then had to recall as much as possible about the story (testing IR). Following a 50-minute retention interval, the subject was again asked to recall anything about the story (testing DR). Finally, the interviewer administered a series of 20 questions (including 15 misleading questions) twice; between the series, he or she gave the subject explicit negative feedback on their performance. Of the 15 misleading questions, 5 were true/false, 5 were forced (with both alternatives false), and 5 were inductive (plausible but leading questions because the story did not provide the answers). The data obtained scores for IR, reflecting the number of distinct ideas (range: 0–40) recalled immediately after the story was read; DR, reflecting the number of distinct ideas recalled after the pause for retention (range: 0–40); Yield 1, the extent to which subjects gave in to the 15 specific questions before receiving negative feedback (range: 0–15); Yield 2, the extent to which subjects gave in to the 15 specific questions after receiving negative feedback (range: 0–15); Shift, the number of distinct response changes to the 20 questions after receiving negative feedback (range: 0–20); and Total Suggestibility, the sum of Yield 1 and Shift scores (range: 0–35). The GSS-2 scales have been shown to demonstrate good reliability and validity [70, 71]. The present study used the Italian version of the test, which showed good internal consistency for all indexes and age groups reported [72]. In the present study, the GSS-2 was used to enable comparisons with previous studies that have investigated suggestibility in the elderly [28–30].

## Data analysis

To verify our first hypothesis, univariate analysis of variance (ANOVAs) tests were run to identify differences between the three groups (late adult, young elderly, and elderly)—which were treated as independent variables—in Interrogative Suggestibility (Total Suggestibility; Yield; Shift), cognitive variables (DR GSS-2 and IR; DR and IR RAVLT; KBIT-2 NV and V) and emotive/affective variables (SES; PSWQ; MCSDS), controlling for years of education. In testing our second and third hypotheses, Pearson’s correlation coefficients (*r*) were calculated to study the associations between Yield scores, Shift scores, and cognitive and emotive/affective variables.

To prove our fourth and fifth hypotheses, moderation analyses and hierarchical regressions were run, respectively. To estimate the impact of the cognitive variables on Yield and the impact of emotive/affective variables on Shift according to the different age groups, moderation models were run using PROCESS version 3.5 [73], as developed by Preacher and Hayes [74] for SPSS, version 25 (IBM, Armonk, NY). A moderator variable is one in which the relationship between the independent and the dependent variable changes across moderator levels, and it is included in statistical models as an interaction term. To assess moderation effects, the relationship between the independent and dependent variable must be significantly different at various levels of the moderator variable [75]. Considering PROCESS model templates [76], we tested a moderation model (Model 1) to explore the moderated effect of age groups on the direct effect of each cognitive variables on Yield and of each emotive/affective variables on Shift, each time controlling for all the others variables. Two separate hierarchical regression models were run to determine the best predictors for Yield and Shift scores, respectively. The first used Yield score as a dependent variable, while the second considered Shift score as a dependent measure. As regards the first model, age and gender were entered in step 1, cognitive variables in step 2, and emotive/affective measures in step 3. For the second model, age and gender were inserted in step 1; MCSDS, SES, and PSWQ in step 2; and DR and IR RAVLT, KBIT-2 V and NV, and IR GSS-2 and DR in step 3.

Finally, ML models were developed to highlight the best strategy for indexing elderly subjects with high suggestibility. ML is a branch of artificial intelligence that enables highly accurate predictions to be made with respect to subject classification. It has recently been introduced in the analysis of real-world datasets [77, 78], including human genetics data [79] and cognitive sciences data [80], as it outperforms traditional statistical methods in terms of model complexity and classification accuracy. For this reason, the present study included ML analysis of the collected data, using WEKA 3.9.

## Results

### Between groups comparison (ANOVAs)

As reported in Table 2, the elderly group demonstrated the worst performance on all GSS-2 variables. In more detail, ANOVAs showed a significant difference with a medium effect size between late adult, young elderly, and elderly groups on GSS-2 measures of Total Suggestibility, Yield, IR, and DR. With respect to Shift scores, the only significant difference was found between the elderly group and the other two groups, with elderly subjects showing the worst performance.

**Table 2:** Between groups comparison (ANOVAs).

		Late adult	Young elderly	Elderly	*F*	*p*	parη^2^
		55–64	65–74	over 75			
		*M* (*SD*)	*M* (*SD*)	*M* (*SD*)			
		*N* = 59	*N* = 63	*N* = 50			
**Interrogative suggestibility variables**							
	**GSS-2 Total Suggestibility**	7.49 (5.92) ^a^	11.30 (5.70) ^b^	15.62 (6.74) ^c^	24.08	<.001	.222
	**GSS-2 Yield**	4.25 (2.96) ^a^	6.81 (3.30) ^b^	8.56 (3.70) ^c^	23.72	<.001	.219
	**GSS-2 Shift**	3.24 (3.52) ^a^	4.49 (3.08) ^a^	7.22 (3.67) ^b^	18.96	<.001	.183
**Cognitive variables**							
	**DR GSS-2**	17.76 (6.45) ^a^	14.33 (6.16) ^b^	10.84 (5.42) ^c^	17.69	<.001	.173
	**IR GSS-2**	20.63 (6.53) ^a^	17.51 (6.26) ^b^	13.84 (4.85) ^c^	17.39	<.001	.171
	**DR RAVLT**	11.21 (2.73) ^a^	10.20 (2.97) ^a^	10.24 (2.70) ^a^	2.40	.094	.028
	**IR RAVLT**	44.14 (7.56) ^a^	42.87 (8.22) ^a^	41.30 (6.26) ^a^	1.95	.145	.023
	**KBIT-2 NV**	99.76 (17.94) ^a^	91.32 (27.71) ^a^	92.26 (19.87) ^a^	2.49	.086	.029
	**KBIT-2 V**	103.66 (14.82) ^a^	99.08 (16.17) ^a^	98.54 (15.99) ^a^	1.85	.160	.021
**Emotive / affective variables**							
	**SES**	23.42 (4.35) ^a^	22.21 (3.69) ^a^	21.62 (4.26) ^a^	2.81	.063	.032
	**PSWQ**	40.52 (17.42) ^a^	45.36 (17.17) ^a^	47.02 (16.71) ^a^	2.18	.116	.025
	**MCSDS**	21.41 (6.26) ^a^	21.86 (4.32) ^a^	23.06 (3.55) ^a^	1.62	.200	.019

*Note*: GSS-2: Gudjonsson Suggestibility Scale-2; IR: Immediate Recall; DR: Delayed Recall; KBIT-2: Kaufman. Brief Intelligence Test-2; RAVLT: Rey Auditory Verbal Learning Test; SES: Rosenberg Self-Esteem Scale; MCSDS: Marlowe–Crowne Social Desirability Scale; PSWQ: Penn State Worry Questionnaire. For each line, different letters indicate a significant difference between columns.

The ANOVAs also showed a non-significant effect on all cognitive and emotive/affective variables (IR RAVLT, DR RAVLT, KBIT-2 NV, KBIT-2 V, SES, PSWQ, and MCSDS) between groups.

### Correlation analysis

Pearson’s correlation analyses (Table 3) showed that Yield scores were significantly negatively correlated with all cognitive variables (IR GSS-2 and DR; IR and DR RAVLT; KBIT-2 NV and V) in all age groups, with the exception of IR and DR RAVLT in the late adult group. With regard to Shift scores, the results showed negative correlations with SES and positive correlations with PSWQ in all three groups. Finally, positive correlations between Shift scores and MCSDS were found in the late adult and young elderly groups, whereas no significant correlation between these factors was found in the elderly group. The magnitude of the correlation coefficients seems to be different in the three groups: this motivated the choice to use age groups as a moderator in the subsequent regression.

**Table 3:** Correlation coefficients (Pearson’s *r*) between GSS-2 Yield and Shift scores and cognitive and emotive/affective variables, respectively.

		Late adult	Young elderly	Elderly
		55–64	65–74	over 75
		*M* (*SD*)	*M* (*SD*)	*M* (*SD*)
		*N* = 59	*N* = 63	*N* = 50
**Yield**				
	**DR GSS-2**	-.553**	-.476**	-.494**
	**IR GSS-2**	-.575**	-.502**	-.409**
	**DR RAVLT**	-.228	-.611**	-.520**
	**IR RAVLT**	-.177	-.448**	-.302*
	**KBIT-2 NV**	-.391**	-.565**	-.554**
	**KBIT-2 V**	-.562**	-.588**	-.407**
**Shift**				
	**SES**	-.394**	-.404**	-.548**
	**PSWQ**	.368**	.321*	.579**
	**MCSDS**	.370**	.550*	-.108

Note:

* p < .05;

** p < .01.

Table A1 in S1 Appendix shows correlation coefficients (Pearson’s *r*) between Yield score and emotive/affective variables; between Shift score and cognitive variables; and between Total Suggestibility score and both cognitive and emotive/affective variables. Table A2 in S1 Appendix, instead, shows correlation coefficients (Pearson’s *r*) between GSS-2 (IR and DR) and (IR and DR) RAVLT, respectively.

### Moderation analyses

Six different moderation models, one for each cognitive variable (KBIT-2 V and NV; IR and DR RAVLT; IR GSS-2 and DR), were examined setting Yield score as the dependent variable. Age groups (late adult, young elderly, elderly) was the moderator. Furthermore, three more moderation models, one for each emotive/affective variable (PSWQ, MCSDS, and SES), were examined setting Shift score as the dependent variable and age groups as the moderator. In each of nine moderation models the others eight variables, both cognitive and emotive/affective, were inserted as covariates.

Not significant interactions were found in moderation models using DR GSS-2, IR GSS-2, IR RAVLT and KBIT-2 V as the dependent variable and Yield as the outcome measure and using SES and PSWQ in moderation models with Shift as the dependent variable. Results showed significant interaction effects in moderation models using DR RAVLT and KBIT-2 NV as the dependent variable and Yield as the dependent measure and using MCSDS with Shift as the dependent variable (Table 4).

**Table 4:** Regression coefficients for moderation models with interactions effects between cognitive and emotive/affective variables and age groups.

**Dependent variable: Yield**	***b***	***SE***	***t***	***p***
**DR GSS-2**	.026	.079	.330	.742
**DR GSS-2 x young elderly**	.-.033	.072	-.455	.650
**DR GSS-2 x elderly**	-.133	.081	-1.646	.102
	*R*^*2*^ = .64 *F*(13, 158) = 21.380***			
**IR GSS-2**	-.108	.078	-1.385	.168
**IR GSS-2 x young elderly**	-.034	.071	-.472	.638
**IR GSS-2 x elderly**	-.077	.086	-.897	.371
	*R*^*2*^ = .63 *F*(13, 158) = 20.961***			
**DR RAVLT**	-.071	.145	-.487	.627
**DR RAVLT x young elderly**	-.109	.164	-.665	.507
**DR RAVLT x elderly**	**-.407**	**.185**	**-2.201**	**.029**
	*R*^*2*^ = .64 *F*(13, 158) = 21.901***			
**IR RAVLT**	.173	.054	3.225	.002
**IR RAVLT x young elderly**	-.051	.060	-.855	.394
**IR RAVLT x elderly**	-.129	.074	-1.749	.082
	*R*^*2*^ = .64 *F*(13, 158) = 21.430***			
**KBIT-2 NV**	-.011	.019	-.599	.550
**KBIT-2 NV x young elderly**	-.024	.022	-1.081	.281
**KBIT-2 NV x elderly**	**-.052**	**.025**	**-2.062**	**.041**
	*R*^*2*^ = .64 *F*(13, 158) = 21.683***			
**KBIT-2 V**	-.042	.023	-1.873	.063
**KBIT-2 V x young elderly**	-.004	.029	-.137	.891
**KBIT-2 V x elderly**	-.005	.031	-.168	.867
	*R*^*2*^ = .63 *F*(13, 158) = 20.798***			
**Dependent variable: Shift**	***b***	***SE***	***t***	***p***
**SES**	-.094	.092	-1.015	.312
**SES x young elderly**	-.012	.127	-.096	.923
**SES x elderly**	-.140	.125	-1.119	.265
	*R*^*2*^ = .53 *F*(13, 158) = 13.652***			
**PSWQ**	.050	.021	2.324	.021
**PSWQ x young elderly**	-.046	.029	-1.562	.120
**PSWQ x elderly**	.028	.031	-.866	.388
	*R*^*2*^ = .54 *F*(13, 158) = 14.347***			
**MCSDS**	.069	.059	1.169	.244
**MCSDS x young elderly**	**-.219**	**.098**	**-2.233**	**.027**
**MCSDS x elderly**	-.203	.123	-1.654	.100
	*R*^*2*^ = .54 *F*(13, 158) = 14.404***			

Note:

*** p < .001. Covariates’ coefficients are not shown.

**Fig 1:**
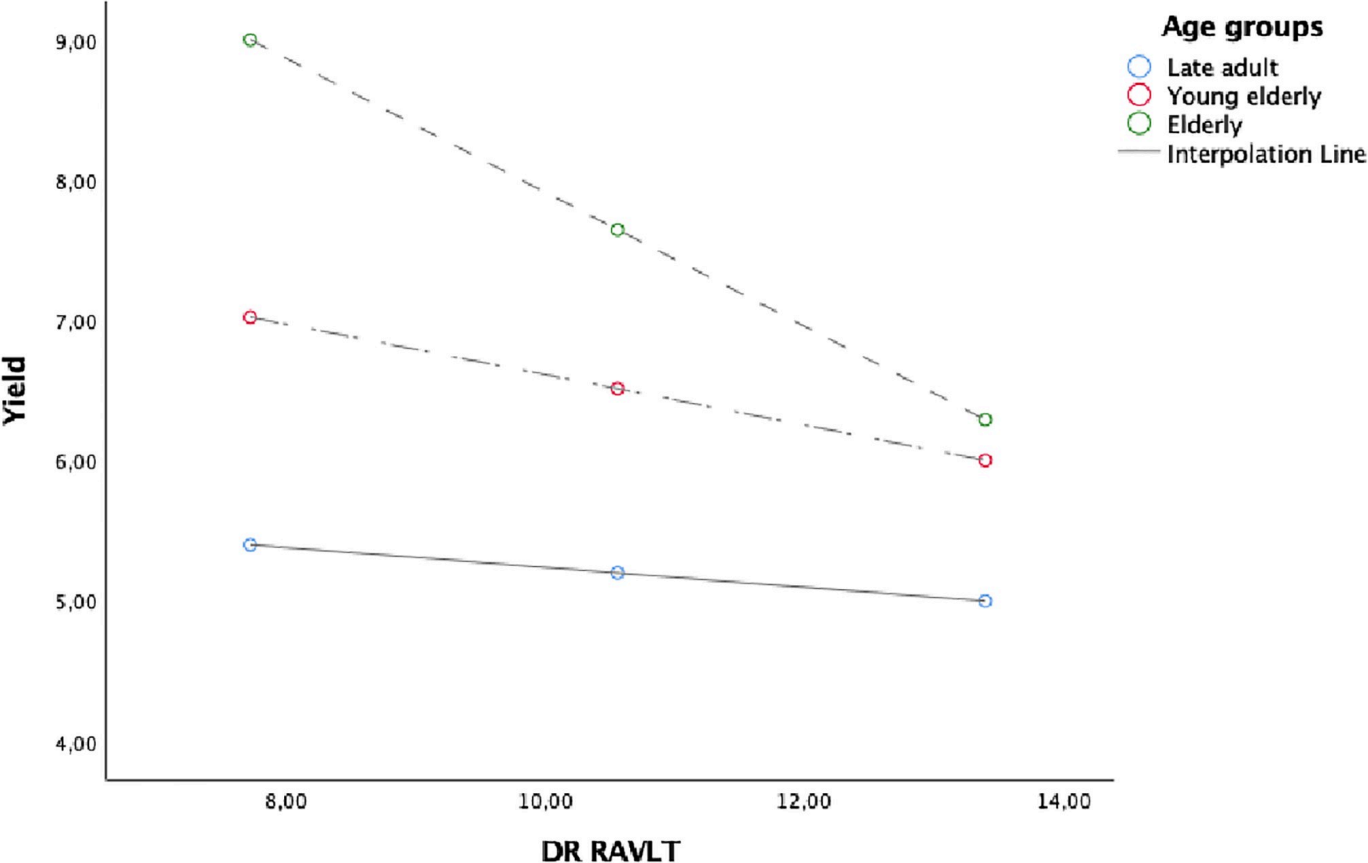
Simple slope analyses with the moderating effect of age groups on the relationship between DR RAVLT and Yield.

**Fig 2:**
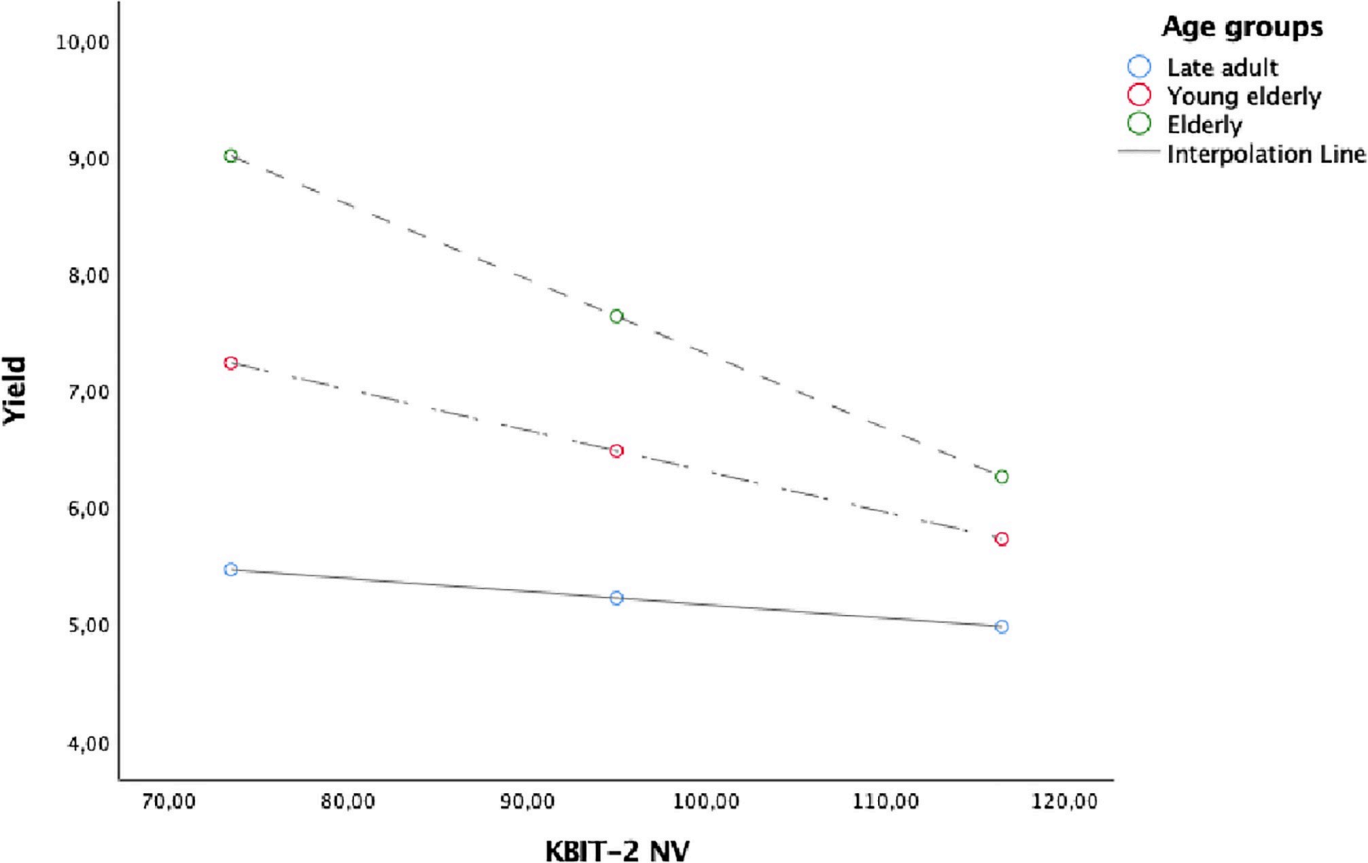
Simple slope analyses with the moderating effect of age groups on the relationship between KBIT-2 NV and Yield.

**Fig 3:**
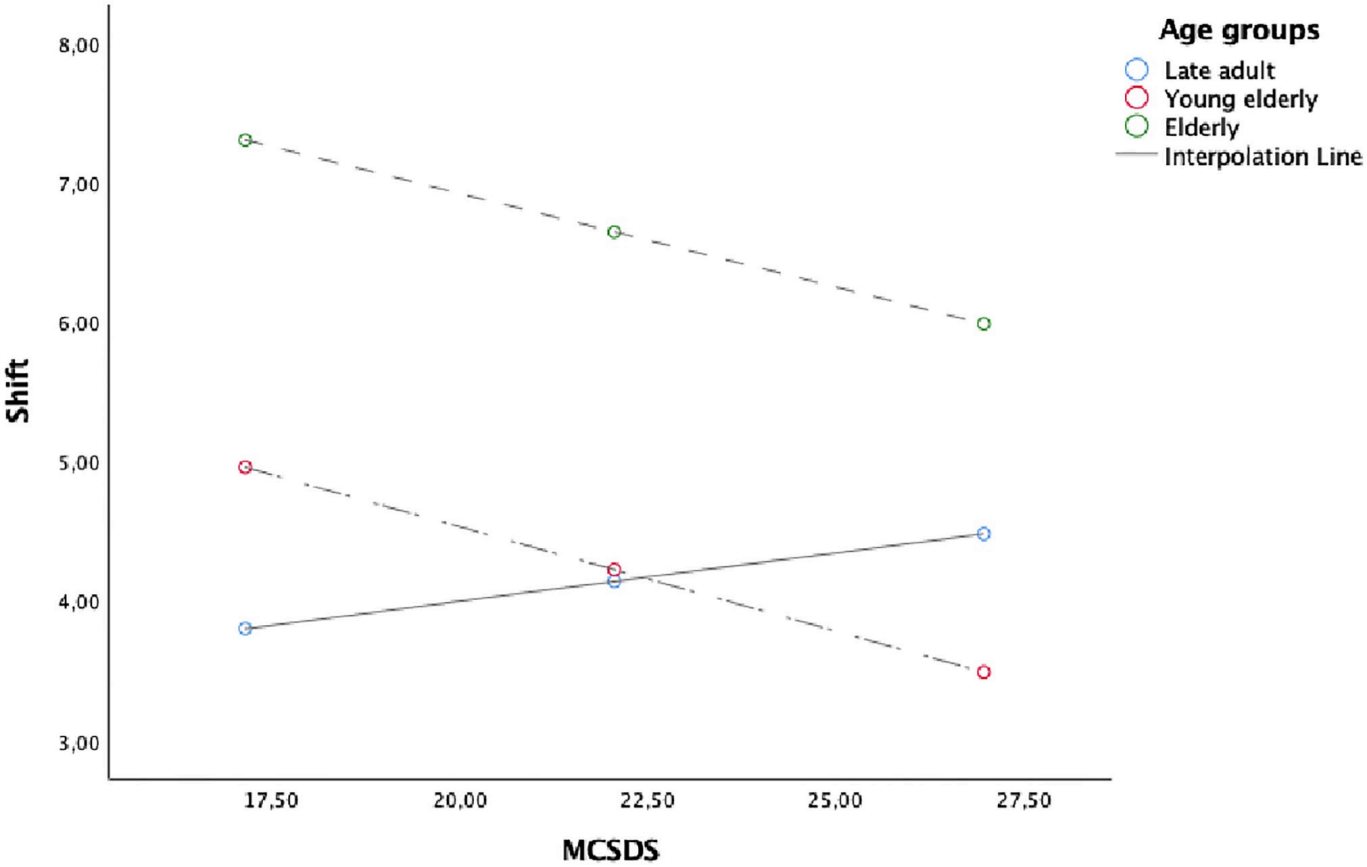
Simple slope analyses with the moderating effect of age groups on the relationship between MCSDS and Shift.

Figs 1, 2 and 3 showed the simple slope analyses with the moderating effect of age groups on the relationship between DR RAVLT and Yield (Fig 1), KBIT-2 NV and Yield (Fig 2) and between MCSDS and Shift (Fig 3).

Age over 75 years (elderly) had an impact on the relationship between DR RAVLT, KBIT-2 NV and Yield. This finding suggests that to be elderly facilitates a negative relationship between the memory and non-verbal intelligence, as measured by DR RAVLT and KBIT-2 NV, and Yield. Furthermore, the negative relationship between social desirability (i.e. MCSDS) and Shift was stronger for those aged between 65–74 (young elderly).

### Regression analysis

A three-step hierarchical multiple regression analysis with the enter method was conducted with Yield score as the dependent variable. Age and gender were entered in step 1 of the regression. Cognitive variables (IR GSS-2 and DR; DR and IR RAVLT; KBIT-2 NV and V) were entered in step 2, and emotive/affective measures (SES; PSWQ; MCSDS) were entered in step 3. The variables were introduced in this order, as Yield score seemed more affected by age and cognitive factors (Table 5).

**Table 5:** Hierarchical linear model of Yield score predictors.

		b	SE B	β	*p*
**Step 1**					
	**Constant**	-6.651	2.28		.004
	**Age**	.194	.033	.412	<.001
	**Gender**	-.362	.520	-.049	.487
**Step 2**					
	**Constant**	9.504	2.276		<.001
	**Age**	.107	.026	.228	<.001
	**Gender**	.448	.390	.060	.253
	**DR GSS-2**	-.065	.068	-.177	.338
	**IR GSS-2**	-.166	.073	-.293	.024
	**DR RAVLT**	-.291	.087	-.222	.001
	**IR RAVLT**	.123	.038	.250	.001
	**KBIT-2 NV**	-.045	.011	-.259	<.001
	**KBIT-2 V**	-.047	.016	-.195	.003
**Step 3**					
	**Constant**	8.899	2.901		.003
	**Age**	.111	.025	.236	<.001
	**Gender**	.481	.377	.065	.204
	**DR GSS-2**	-.055	.066	-.098	.405
	**IR GSS-2**	-.126	.072	-.222	.080
	**DR RAVLT**	-.266	.085	-.204	.002
	**IR RAVLT**	.119	.037	.240	.002
	**KBIT-2 NV**	-.040	.011	-.234	<.001
	**KBIT-2 V**	-.041	.015	-.172	.008
	**SES**	-.113	.054	-.126	.038
	**PSWQ**	.033	.012	.151	.008
	**MCSDS**	-.020	.039	-.027	.606

*Note*: R^2^ = .17 for step 1; ΔR^2^ = .17. R^2^ = .59 for step 2; ΔR^2^ = .42. R^2^ = .63 for step 3; ΔR^2^ = .04.

The hierarchical multiple regression revealed that, at step 1, age contributed significantly to the model, *F* (2,169) = 17.44, *p* < .001, accounting for 17% of the variation in Yield scores. Introducing the cognitive variables explained an additional 42% of the variation in Yield scores, and this change in *R*^*2*^ was significant, *F* (8,163) = 29.27, *p* < .001. Finally, adding emotive/affective measures to the regression model explained an additional 4% of the variation in Yield scores, and this change in *R*^*2*^ was also significant, *F* (11,160) = 24.68, *p* < .001. When all 11 independent variables were included in step 3, the predictive values of gender, IR GSS-2 and DR, and MCSDS were all non-significant. The most important predictors of Yield score were age, IR RAVLT, and KBIT-2 NV, which explained 24%, 24%, and 23% of the variation in Yield scores, respectively. Together, the 11 independent variables accounted for 60% of the variance in Yield scores.

Following this analysis, a three-step hierarchical multiple regression with the enter method was conducted with Shift score as the dependent variable. Age and gender were entered in step 1, emotive/affective measures (SES; PSWQ; MCSDS) were entered in step 2, and cognitive variables (IR GSS-2 and DR; DR and IR RAVLT; KBIT-2 NV and V) were entered in step 3. The variables were introduced in this order because Shift score seemed more affected by age and emotive/affective factors (Table 6).

**Table 6:** Hierarchical linear model of shift score predictors.

		b	SE B	β	*p*
**Step 1**					
	**Constant**	-6.64	2.36		.006
	**Age**	.170	.034	.357	<.001
	**Gender**	-.358	.540	-.048	.508
**Step 2**					
	**Constant**	-1.235	3.211		.701
	**Age**	.139	.030	.291	<.001
	**Gender**	-.024	.472	-.003	.960
	**SES**	-.266	.066	-.293	<.001
	**PSWQ**	.061	.015	.281	<.001
	**MCSDS**	-.005	.050	-.007	.920
**Step 3**					
	**Constant**	7.098	3.410		.039
	**Age**	.102	.029	.215	.001
	**Gender**	.255	.443	.034	.566
	**SES**	-.125	.063	-.138	.050
	**PSWQ**	.042	.014	.193	.004
	**MCSDS**	-.018	.046	-.023	.705
	**DR GSS-2**	-.030	.077	.053	.698
	**IR GSS-2**	-.162	.084	-.282	.056
	**DR RAVLT**	-.124	.100	-.094	.216
	**IR RAVLT**	.015	.043	.030	.727
	**KBIT-2 NV**	-.040	.013	-.228	.002
	**KBIT-2 V**	-.012	.018	-.048	.517

*Note*: R^2^ = .13 for Step 1; ΔR^2^ = .13. R^2^ = .36 for Step 2; ΔR^2^ = .23. R^2^ = .50 for Step 3; ΔR^2^ = .14.

The hierarchical multiple regression revealed that, at step 1, age contributed significantly to the model, *F* (2,169) = 12.55, *p* < .001, accounting for 13% of the variation in Shift scores. Introducing the emotive/affective variables explained an additional 23% of the variation in Shift scores, and this change in *R*^*2*^ was significant, *F* (5,166) = 18.72, *p* < .001. Finally, adding cognitive measures to the model explained an additional 14% of the variation in Shift scores, and this change in *R*^*2*^ was also significant, *F* (11,160) = 14.60, *p* < .001. When all 11 independent variables were included in step 3, gender, MCSDS, IR GSS-2 and DR, IR and DR RAVLT, and KBIT-2 V were all found to be non-significant predictors of Shift score. The most important predictors of Shift score were age and KBIT-2 NV, which explained 22% and 23% of the variation in Shift scores, respectively. Together, the 11 independent variables accounted for 47% of the variance in Shift scores.

In the following analyses, suggestibility was divided into two subgroups of high and low suggestibility. Rather than addressing the correlation with test scores, the classification of subjects into high and low suggestibility groups was investigated, leading to a determination of the diagnostic conditions of high suggestibility.

### Machine learning models

In the last years, there is increasing interest in complementing the statistical explanatory approach with a prediction approach [53, 81]. For this reason, the present study tested the accuracy of the variables highlighted in the previous section by applying appropriate ML models to identify the overall predictive accuracy of suggestibility indexes on the basis of cognitive and emotive/affective variables. The final goal of using ML is to find a model that best generalizes to new unseen data, avoiding data overfitting. Indeed, compared to the traditional analysis, ML introduces a series of strategies to improve generalization and reduce overfitting, such as the cross-validation technique. Moreover, being a data driven approach, ML allows the automated selection of features (feature engineering), squeezing as much predictive power as possible out of a model, using whichever combination of features does that. Finally, ML gives the opportunity to use complex algorithms to identify tricky relations in the data, classifying also non-linear separable data.

As a preliminary step, the original sample of participants was split into two groups of high and low suggestibility, on the basis of their GSS-2 Total Suggestibility scores (Yield+Shift scores). The lowest scoring 51 participants (out of the total 172) comprised the low suggestibility group while the highest scoring 52 participants (out of the total 172) comprised the high suggestibility group. Average Yield, Shift, and Total Suggestibility scores of the low and high suggestibility groups are reported in Table 7.

**Table 7:** Average scores in Yield, Shift, and Total Suggestibility of low and high suggestibility groups.

		Low	High
		(*N* = 51)	(*N* = 52)
**GSS-2 Yield**	*M*(*SD*)	2.35 (1.38)	10.48 (1.95)
**GSS-2 Shift**	*M*(*SD*)	0.99 (1.12)	9.00 (2.27)
**GSS-2 Total Suggestibility**	*M*(*SD*)	3.29 (1.62)	19.62 (3.01)

A correlation-based analysis was conducted to investigate the relative contribution of each independent variable [53]. A preliminary analysis indicated that the GSS-2 Total Suggestibility score (high vs. low) was most strongly associated with KBIT-2 NV (0.72), IR GSS-2 (0.65), KBIT-2 V (0.64), and DR GSS-2 (0.63). However, given the high correlations (r_pb_) found between the independent variables within the sample, all independent variables were included in the analysis. Finally, ML algorithms were trained using a 10-fold cross-validation procedure, as follow: first, the sample of participants was randomly partitioned into 10 equal subsamples. One of the 10 subsamples was retained as the validation set for model testing and the remaining 9 subsamples were used as training sets. This cross-validation process was recursively repeated 10 times. Finally, the 10 results from the folds were averaged to produce a single estimate of classification accuracy. The accuracies obtained by the four ML classifiers (Logistics, Naïve Bayes, SVM, and Random Forest) trained on the dataset of high and low suggestibility participants fell in the range of 86–90% (Table 8). It should be noted that the classification accuracies were stable across different classifiers, showing that the results did not depend on the specific assumptions made by each model. In fact, the five classifiers were representative of differing underlying classification strategies.

**Table 8:** Classification metrics of ML algorithms developed on the low and high suggestibility samples.

ML classifier	Accuracy	AUC	False positive	False negative
Naive Bayes	90%	0.94	7/51	2/52
Logistics	86%	0.85	8/51	6/52
SVM	89%	0.89	7/51	4/52
Random Forest	88%	0.94	8/51	4/52
OneR	82%	0.83	8/51	10/52

*Note*: False positive = low suggestibility classified as high; false negative = High suggestibility classified as low. AUC = area under the curve.

Results from ML models (such as those reported above) are often difficult to interpret. Indeed, in the present study, the precise mechanics that yielded the algorithm that categorized participants into groups was unclear. For this reason, to better understand the decision rules on which the classification results were based, the most accurate diagnostic decision rule of the 10 ML models was identified using the OneR algorithm, as implemented in WEKA 3.9 [54]. This algorithm showed that the most accurate diagnostic classification rule placed subjects in the high suggestibility group on the basis of a IR GSS-2 score equal to or less than 18.5 (L = 0.19; H = 0.8) and in the low suggestibility group on the basis of a score higher than 18.5 (L = 0.98; H = 0.02). This rule correctly classified 85 (out of 103) subjects, generating an accuracy of 82% and an AUC value of .83.

Table 8 reports the classification accuracy and other relevant metrics. The reported classifiers belong to different classes and are reported in order to index that the high accuracy did not result from specific assumptions.

Finally, two separate analyses were conducted on Shift and Yield scores, respectively. Similarly to the previously reported analyses, high (*n* = 50) versus low (*n* = 50) Shift scoring participants were analyzed. Shift score showed maximum correlation with IR GSS-2 (0.6457), DR GSS-2 (0.59), KBIT-2 NV (0.5758), and SES (0.51). An interpretable classifier, OneR, correctly classified 85 out of 101 instances according to the following rule: if IR GSS-2 < 20, then high Shift score (else low Shift score). The least accurate classifier was generated by Logistics (79%) and the highest was generated by Naïve Bayes (88%).

The corresponding analysis of Yield scores (contrasting 50 high with 50 low Yield scoring participants) showed that the highest correlations were with KBIT-2 NV (0.69), DR GSS-2 (0.66), IR GSS-2 (0.66), and KBIT-2 V (0.6). Again, a single interpretable decision rule (if IR GSS-2 < = 20.5, then high Yield score [else low Yield score]) correctly classified 83% of the instances.

Taken together, the ML analysis indicated that IR GSS-2 correctly classified more than 80% of the participants. This classification accuracy regarded Total Suggestibility, Shift, and Yield scores. Thus, in general, indices of memory performance (IR GSS-2) and cognitive level (KBIT-2 V) may be used to predict—with high accuracy—the suggestibility of elderly persons.

## Discussion

In order to improve knowledge about IS in the elderly, the present study investigated suggestibility in the elderly compared to younger adults. The results showed a progressive decline in suggestibility, with older participants more suggestible compared to the late adult and young elderly groups.

The results confirmed the first hypothesis, showing that the elderly had the worst performance on all GSS-2 indexes. These data are aligned with the previous studies of Mueller-Johnson and Ceci [29] and Dukala and Polczyk [28], which found higher levels of suggestibility in older participants (albeit their samples were younger than that of the present study). These previous studies also highlighted a relationship between age and the tendency to yield to suggestive questions, mediated by the quality of memory [28, 30, 82]. Overall, memory quality decreases with increasing age, indicating a worsening trend in performance on all memory-related variables, including IR GSS-2 and DR. Specifically, in the present study, the IR GSS-2 and DR scores of the elderly group showed lower memory—and reduced recall efficiency—relative to the other (younger) groups, in line with previous studies [28, 83]. The results also showed a statistically significant difference between the elderly group and the late adult and young elderly groups in the Shift scale, which is more associated with interpersonal and psychosocial variables than with memory [28, 30, 84].

The results also confirmed the second hypothesis, showing that low levels of memory and intelligence were correlated with high Yield scores. Consistent with previous studies [28–30, 34, 35, 85–90], the present study found that elderly participants scored lower on memory and intelligence measures and showed a related poor performance on IS variables. These findings confirm the association—well-known in the literature [83, for a review]—between Yield score and cognitive factors, underlining that older people tend to have reduced memory and greater suggestibility than younger persons, due to a cognitive ability and a tendency to become more confused and less certain following misleading questions [18, 37, 91, 92].

The third hypothesis was mostly confirmed by the results, which underlined a positive relation between Shift score and worry and a negative relation between Shift score and self-esteem. This finding supports the idea that social factors have a prevalent influence on IS [83, for a review]. In more detail, worry—a component of state anxiety and the main cause of its negative effects [93]—was found to be positively correlated with high suggestibility [16, 18, 94]: by reducing cognitive resources [93], worry may have increased suggestibility, especially following negative feedback [95], leading to higher Shift scores. Shift was also found to be negatively correlated with self-esteem [22, 23, 26, 96]—a factor that may affect one’s self-evaluation of readiness to face an interview situation, influencing the subject’s suggestibility, especially in the context of negative feedback [96–98]. Positive correlations between Shift scores and social desirability were found only in the late adult and young elderly groups. This was an unexpected outcome, in light of the results of previous studies [11, 21, 88, 99], but it may relate to the mean age of the participants in the elderly group of the present research, which was higher than that of previous studies. A lower influence of social desirability in older people (relative to younger ones) was underlined by Fastame, Penna, and Hitchcott [95], who found this variable to play a marginal role in predicting the psychological well-being of the elderly, confirming the findings of Phillips, Henry, Hoise, and Milne [100]. Moderation analysis also confirmed this finding, showing a stronger impact of the social desirability on Shift scores for those aged between 65 to 74 years old.

The present study also explored the possibility of using age and cognitive variables to predict Yield scores, together with the possibility of using age and emotive/affective variables to predict Shift scores. Results showed that age was the most important predictor of both Yield and Shift and confirmed the important role of intelligence in its non-verbal form (as measured by KBIT-2 NV) and memory (as measured by IR RAVLT and DR RAVLT) in explaining subject’s tendency to yield to misleading questions, again in line with previous researches [10, 18, 32, 101]. Moderation results showed that this association was stronger for those aged over 75 years. It is noteworthy that the best predictor of Shift resulted to be the non-verbal intelligence (as measured by KBIT-2 NV), together with age, even though the well-known relation between this IS variable and social factors [83, for a review]. Self-esteem and worry, indeed, even if significant, explained a minor percentage of the variation in Shift. Since none of the previous studies on IS in elderly [28–30] administered the KBIT-2, it is important to investigate whether the structure of this specific test could affect Shift scores, even considering the significant negative correlation found between these two measures. Indeed, the KBIT-2 is not a self-administered test, therefore it is possible the influence of the interviewer’s behavior on subject’s attitude toward the test and its administration.

In line with the recent emphasis on replicability highlighting the need for behavioral prediction (beyond mere statistical analysis), the present study developed predictive ML models to estimate the maximum accuracy with which participants with low versus high suggestibility could be predicted. All ML models distinguished between the two classes (high vs. low suggestibility) with accuracy ranging from 86–90% and AUC values above 0.85. While most ML models operated as efficient classifiers, they were difficult to interpret. To engender greater insight into the rules used to distinguish low versus high suggestibility, an additional model was used to identify the single best classification rule. This model demonstrated that IR GSS-2 classified 82% of participants correctly. More specifically, IR GSS-2 scores below 18.5 indexed high suggestibility, while scores above 18.5 indexed low suggestibility. This efficient classification rule indicated that participants with low verbal recall were also high in suggestibility; in other words, memory impairment was found to be the best predictor of suggestibility [32]. Similar results were observed in predicting Shift and Yield scores. A possible explanation for the predictive capacity of immediate recall on suggestibility is that resistance to suggestion requires strong memory; thus, poor recall may leave subjects more vulnerable to the influence of suggestion. This result is of great importance to medico-legal settings, in which the mental capacity of older persons to resist undue influences is evaluated. Thus, the result reported here has practical consequences for the evaluation of natural capacities and protection issues [85].

## Strengths and limitations

The present study aimed at contributing to a better understanding of IS in the elderly, considering the abovementioned increase in the number of senior citizens in most nations and the lack of research on older samples. It generated useful insight into the phenomenon of IS in the elderly, examining variables that had not been previously considered in the literature (e.g., worry, self-esteem, verbal memory, verbal skills, and logical abilities) in subjects aged over 75 years. Nevertheless, there are some important limitations of this study that require supplementary research to overcome: (a) the GSS-2, which is highly sensitive to individual differences between interviewers, was administered by two different interviewers (though both were trained by experts and aligned in their interpretation and scoring); (b) Confabulation—an important GSS-2 index that was previously studied by Gudjonsson [83, 102] and Sigurdsson et al. [103]—was not taken into account; (c) there were significant differences in the years of education between the study sample groups, and these differences may have affected IS scores; (d) the research design was cross-sectional: we could not follow the changes in suggestibility over time. Results emerging from cross-sectional plans are, indeed, weaker compared to those that could raise from longitudinal researches; e) the present study does not compare the results of this specific sample (aged between 55 and 86 years) with a younger one. So, it is not possible to determine to what extent the performance of the late adult participants on all the variables considered differs from the performance of a younger sample.

In the interest of improving our understanding of IS, future research should extend the hypotheses and sample used in the present work. An additional study should investigate and measure participants’ memory using the WMS, which is frequently applied in studies with younger adults. It would also be useful to investigate delayed IS [90, 97] by administrating GSS-2 questioning right after the IR—obtaining Yield, Shift, and Total Suggestibility scores—and DR after 1 week, in order to evaluate the tendency to incorporate into original memory the information provided by suggestive questions. In legal contexts, the importance of identifying the characteristics of both victims and perpetrators is clear. Future research, therefore, should take into account the contributions of Bain and Baxter [98] and Dukala and Polczyk [28], who studied interviewer behavior as a predictor of IS, finding that older people interviewed under abrupt (vs. friendly) conditions scored higher on Shift relative to younger persons.

## Conclusions

Although further research is required to achieve a comprehensive view of IS in the elderly, the results of the present study suggest that several variables are involved. In particular, the results confirmed the initial hypotheses that suggestibility increases with age and that it is correlated with cognitive variables—specifically intelligence and memory, in line with the results of previous studies. The present research demonstrated that IS in the elderly is also correlated with emotive/affective variables, such as worry and self-esteem, confirming its multifaceted nature. This finding was confirmed by the ML analysis [104, 105], which underlined the importance of memory (predominantly) and all of the studied cognitive and emotive/affective variables in explaining IS in an elderly population.

Overall, despite the possible limitations of the present study, it provided new data on IS, clarifying the influence of cognitive and emotive/affective variables on the ability to withstand suggestive questions. Most importantly, the research considered subjects over the age of 75, who had never been considered empirically in this context, but who hold an increasingly prevalent role in the forensic field.

## Supporting information

S1 Appendix (DOCX)
