# Supplementary material for: Interrogative suggestibility in the elderly
Source: PLoS One. 2020 Nov 16;15(11):e0241353. doi: 10.1371/journal.pone.0241353 (PMC7668574; doi:10.1371/journal.pone.0241353)
Supplement: S1 Appendix — (DOCX) [file pone.0241353.s001.docx]

**S1 Appendix**

**Table A1. Correlation coefficients (Pearson’s *r*) between Yield score and emotive/affective variables; between Shift score and cognitive variables; and between Total Suggestibility score and both cognitive and emotive/affective variables.**

|  |  | **55–64** | **65–74** | **over 75** |
| --- | --- | --- | --- | --- |
|  |  | ***M* (*SD*)** | ***M* (*SD*)** | ***M* (*SD*)** |
|  |  | ***N* = 59** | ***N* = 63** | ***N* = 50** |
| **Yield** |  |  |  |  |
|  | **SES** | -.421^**^ | -.481^**^ | -.484^**^ |
|  | **MCSDS** | -.256 | .009 | -.014 |
|  | **PSWQ** | .228 | .467^**^ | .482^**^ |
| **Shift** |  |  |  |  |
|  | **IR GSS-2** | -.597^**^ | -.461^**^ | -.329^**^ |
|  | **DR GSS-2** | -.552^**^ | -.403^**^ | -.332^**^ |
|  | **DR RAVLT** | -.427^**^ | -.383^**^ | -.381^**^ |
|  | **IR RAVLT** | -.367^**^ | -.480^**^ | -.291^*^ |
|  | **KBIT-2 NV IQ** | -.241 | -.596^**^ | -.549^**^ |
|  | **KBIT-2 V IQ** | -.407^**^ | -.421^**^ | -.373^**^ |
| **Total Suggestibility** |  |  |  |  |
|  | **IR GSS-2** | -.644^**^ | -.540^**^ | -.396^**^ |
|  | **DR GSS-2** | -.606^**^ | -.494^**^ | -.452^**^ |
|  | **IR RAVLT** | -.308^*^ | -.518^**^ | -.258 |
|  | **DR RAVLT** | -.369^**^ | -.561^**^ | -.474^**^ |
|  | **KBIT-2 V IQ** | -.524^**^ | -.568^**^ | -.435^**^ |
|  | **KBIT-2 NV IQ** | -.340^**^ | -.649^**^ | -.591^**^ |
|  | **SES** | -.446^*^ | -.496^**^ | -.590^**^ |
|  | **MCSDS** | .349^**^ | .200 | -.066 |
|  | **PSWQ** | .334^**^ | .443^**^ | .599^**^ |

*Note:* * p < .05; ** p < .01.

**Table A2. Correlation coefficients (Pearson’s *r*) between GSS-2 (IR and DR) and (IR and DR) RAVLT, respectively.**

|  |  | **55–64** | **65–74** | **over 75** |
| --- | --- | --- | --- | --- |
|  |  | ***M* (*SD*)** | ***M* (*SD*)** | ***M* (*SD*)** |
|  |  | ***N* = 59** | ***N* = 63** | ***N* = 50** |
| **IR GSS-2** |  |  |  |  |
|  | **IR RAVLT** | .530^**^ | .826^**^ | .679^**^ |
|  | **DR RAVLT** | .590^**^ | .531^**^ | .273 |
| **DR GSS-2** |  |  |  |  |
|  | **IR RAVLT** | .416^**^ | .764^**^ | .565^**^ |
|  | **DR RAVLT** | .519^**^ | .598^**^ | .402^**^ |

*Note:* ** p < .01.
